# Are they functional hypogonadal men? Testosterone serum levels unravel male idiopathic infertility subgroups

**DOI:** 10.1007/s12020-024-03717-3

**Published:** 2024-02-19

**Authors:** Giorgia Spaggiari, Francesco Costantino, Leonardo Dalla Valentina, Marilina Romeo, Chiara Furini, Laura Roli, Maria Cristina De Santis, Giulia Canu, Tommaso Trenti, Antonio R. M. Granata, Manuela Simoni, Daniele Santi

**Affiliations:** 1 Unit of Endocrinology, Department of Medical Specialties, Azienda Ospedaliero-Universitaria of Modena, Modena, Italy; 2 Unit of Andrology and Sexual Medicine of the Unit of Endocrinology, Department of Medical Specialties, Azienda Ospedaliero-Universitaria of Modena, Modena, Italy; 3 Department of Biomedical, Metabolic and Neural Sciences, University of Modena and Reggio Emilia, Modena, Italy; 4 Department of Laboratory Medicine and Pathology, Azienda USL of Modena, Modena, Italy

**Keywords:** Testosterone, Male infertility, Idiopathic, FSH, Hypogonadism

## Abstract

**Purpose:**

To evaluate total testosterone distribution in male idiopathic infertility.

**Methods:**

A retrospective, real-world case-control clinical study was conducted. Cases consisted of men evaluated for couple infertility, specifically those with alterations in semen parameters and normal gonadotropin levels, and after excluding all known causes of male infertility. Controls were male subjects who underwent semen analysis for screening purposes, without any abnormality detected. The total testosterone distribution was evaluated in cases and controls. Further analyses were performed subgrouping cases according to total testosterone reference threshold suggested by scientific societies (i.e., 3.5 ng/mL).

**Results:**

Cases included 214 idiopathic infertile men (mean age 38.2 ± 6.2 years) and controls 224 subjects with normozoospermia (mean age 33.7 ± 7.5 years). Total testosterone was not-normally distributed in both cases and controls, with positive asymmetric distribution slightly shifted on the left in cases. The rate of subjects with testosterone lower than 3.5 ng/mL was higher in cases (23.8%) than controls (4.5%) (*p* < 0.001). In cases with testosterone lower than 3.5 ng/mL, a significant direct correlation between testosterone and the percentage of normal morphology sperms was highlighted, also applying multivariate stepwise linear regression analysis (*R* = 0.430, standard error = 0.3, *p* = 0.020).

**Conclusion:**

Although idiopathic infertile men show by definition altered semen analysis and gonadotropins within reference ranges, testosterone serum levels are widely variable in this population. Approximately a quarter of these patients present some sort of functional hypogonadism. Our data support the need to better classify idiopathic male infertility and total testosterone serum levels could be a supportive parameter in tracing the patient’s therapeutic profile.

## Introduction

Male infertility is estimated to affect about 50% of all reproductive-aged infertile couples in Western countries [1, 2]. In about 40% of the cases, the underlying cause of male infertility could not be identified, falling into the broad category of male idiopathic infertility. By definition, this condition is characterized by at least one altered semen parameter according to World Health Organization (WHO)’s decision limits, in the absence of any recognizable cause [3]. Since etiological factors remain unknown, proposed therapies in idiopathic infertility setting are empirical. Among potential treatments, the empirical testicular stimulation through exogenous follicle-stimulating hormone (FSH) has been reported in the literature. The comprehensive evaluation of clinical trials designed to investigate FSH efficacy in male idiopathic infertility showed an overall increased pregnancy rate after treatment, burdened by a high number-need-to-treat to obtain a single pregnancy [4, 5]. This evidence could have two different interpretations: from one side, FSH could be evaluated as essentially ineffective in case of male idiopathic infertility, while, on the other side, FSH could be effective, but the current treatment scheme should be optimized in terms of dose and duration. Indeed, a recent real-world study suggested that the actual FSH therapy leads to a 27.6% pregnancy rate when administered to idiopathic infertile men [6], while a dose-dependent FSH efficacy on semen parameters has been suggested in the literature [7, 8]. In this context, one of the crucial points remains the need to recognize a priori FSH-responders and consequently to have the possibility to tailor the FSH scheme according to patients’ characteristics.

Few attempts have been performed so far to stratify patients a priori, using pharmacogenetics predictors of FSH efficacy [9–11]. However, conflicting results have been obtained and no validated strategies to personalize hormonal treatment in male idiopathic infertility are available nowadays. Surely, the genetic background of idiopathic infertile men must be considered. Several authors suggested that male idiopathic infertility is caused by not yet identified genetic abnormalities acting alone or in multiple combinations [12]. Accordingly, male idiopathic infertility is a large and heterogeneous diagnostic definition that includes conditions extremely different from each other. Male idiopathic infertility is not a single entity but the sum of several subgroups, each one with a possible different response to exogenous FSH administration. From a practical point of view, male idiopathic infertility has been considered as a form of functional hypogonadotropic hypogonadism, a condition in which gonadotropin serum levels are ‘inappropriately’ within reference ranges, while the target gland does not respond optimally. According to that and mimicking the therapeutic approach used in hypogonadotropic hypogonadism, FSH is proposed to idiopathic infertile men as a hormonal replacement therapy. On the other hand, other authors suggested that male idiopathic infertility is not classifiable as alternative form of hypogonadotropic hypogonadism, since gonadotropins’ activity is present and not absent as seen in hypogonadotropic hypogonadism. According to these considerations, exogenous FSH administration could be proposed with a stimulatory aim, instead of a replacement one. Clearly, if it was possible to clarify whether idiopathic infertility is or not a form of hypogonadotropic hypogonadism, this would have repercussions in therapeutic management, supporting a stimulatory *versus* substitutive approach.

It is well known that high levels of intratesticular testosterone are required to support a qualitatively and quantitatively normal spermatogenesis [13–16]. Accordingly, the European Association of Urology (EAU) [17] and the Italian Society of Andrology and Sexual Medicine (SIAMS) [18] recommended at least one testosterone serum levels measurement in infertile men. This is physiologically appropriate, since it allows to evaluate the testicular function as a whole. But are testosterone serum levels useful to discriminate between functional hypogonadotropic hypogonadism to other conditions resulting in male idiopathic infertility? The distribution of testosterone serum levels in men with idiopathic infertility has been evaluated in old studies, providing conflicting results [19–31]. With this in mind, the aim of the study was the evaluation of testosterone serum levels distribution in a cohort of men with idiopathic infertility to possibly clarify its clinical implications. Moreover, idiopathic infertile men were compared to controls.

## Materials and methods

### Study design

A retrospective, observational case-control clinical study was carried out based on real-world data.

Cases consisted of all patients evaluated at the Andrology Unit for couple infertility issues from June 2016 to June 2023. Each patient underwent the standard diagnostic work-up provided for male infertility [32], as already described elsewhere [33]. Thus, history collection, physical examination, semen analysis and hormonal evaluations were performed and collected. Only those patients entering the diagnostic class of male idiopathic infertility were finally enrolled in the study. Thus, each patient was evaluated more than once, in order to exclude all known causes of male infertility. In particular, the following inclusion criteria were considered for cases: (i) male partner of infertile couples (i.e., couples who did not reach a pregnancy after at least 12 months of unprotected sexual intercourses), (ii) with alteration in at least one of semen analysis parameter, (iii) with normal FSH (range 1–12 IU/L) and luteinizing hormone (LH) (range 1–9 IU/L) serum levels, and (iv) with total testosterone serum levels higher than 2.1 ng/mL. The reference ranges of FSH and LH serum levels were those suggested by the kit used to perform the examination by the laboratory. On the contrary, reference ranges for testosterone serum levels were not those suggested by laboratory kit. Indeed, a widely accepted testosterone threshold for the definition of hypogonadism is still lacking. Considering scientific guidelines on the definition of clinical hypogonadism [34, 35], testosterone serum levels lower than 2.1 ng/mL surely recognize hypogonadal men, while values higher than 3.5 ng/mL identify eugonadal ones. With this in mind, we excluded men in which the diagnosis of hypogonadism was reached beyond any doubt, thus when testosterone serum levels were <2.1 ng/mL. Alongside hypogonadism, known and demonstrated causes of infertility were excluded, such as the genetic alterations (i.e., chromosomal alterations/aberrations, Y-chromosome microdeletions and cystic fibrosis transmembrane conductance regulator [CFTR] gene mutations) presence of varicocele, urogenital infections, obstructive forms of infertility, other endocrinopaties potentially affecting the gonadal function (i.e., Cushing syndrome, pituitary tumours, adrenal gland dysfunctions).

Controls included all men consecutively referred to the Department of Laboratory Medicine and Pathological Anatomy, Azienda USL of Modena, from September 2010 to May 2022 for semen analysis for screening purpose. Subjects were extracted and identified by an anonymous unique personal alphanumeric code, as published elsewhere [36]. A single dataset was generated, including patient’s age, LH, FSH, testosterone and prolactin serum levels and conventional semen analysis. Starting from this dataset, only subjects with normozoospermia were considered. In details, the definition of normozoospermia required all the following criteria satisfied: (i) sperm concentration higher than 16 million/mL, (ii) total sperm number higher than 39 million, (iii) progressive sperm motility higher than 30%, and (iv) normal sperm morphology higher than 4% [37].

### Data collected

For cases, the final dataset included history of couple infertility (i.e., partner’s age, duration of infertility, primary or secondary infertility, previous or current treatment for male infertility), personal history (including comorbidities, number of drugs, chronic disease score [CDS]), physical examination (body mass index [BMI], testicular volume measured at orchidometer and at testicular ultrasound examination), hormone serum levels (LH, FSH, total testosterone, estradiol and prolactin) and semen analysis. The CDS was calculated based on the current medication use returning an aggregate comorbidity measure [38, 39]. In particular, 25 classes of medication are weighted in the total CDS score reflecting the disease complexity and severity [39]. Higher CDS values correspond to more severe comorbidity status (range 0–35) [38].

For controls, the final database included LH, FSH, total testosterone, prolactin and semen analysis.

For both cases and controls, conventional semen analysis was performed at the Department of Laboratory Medicine and Pathology, Azienda USL of Modena on a semen sample collected through masturbation after 2–7 days of sexual abstinence. Among sperm parameters, the following variables were considered: semen volume, pH, sperm concentration and total number, sperm progressive and total motilities and normal sperm morphology. The analysis was performed according to V edition of WHO manual, until 2021 and following the VI edition in the last two years [37]. Blood samples were obtained after an overnight fast, in the morning (8.00 am) to evaluate hormonal data. Total testosterone serum levels were assayed by Chemiluminescent Microparticle Immunoassay (Achitect, Abbott, Dundee, UK). LH, FSH and estradiol were measured by ARCHITECT platform (Abbott Laboratories, USA). Prolactin was measured by Chemiluminescent Immunoassay (Beckman Coulter, Brea, CA, USA). The testosterone on LH ratio (T/LH) was calculated as potential independent predictor of spermatogenesis [40].

### Ethical

The study protocol was approved by the local ethics committee of the “Area Vasta Emilia Nord Modena” (protocol number AOU0024637/19 of 09/2019). Due to the retrospective design of the study, informed consent was not necessary.

### Statistical analysis

Data distribution was evaluated with Shapiro–Wilk test. Cases and controls data distribution was compared applying Mann–Whitney *U*-test.

Total testosterone serum levels distribution was described and the confidence interval at 95% was calculated. Then, sub-analyses were performed among cases, using the threshold of 3.5 ng/mL to define normal testosterone serum levels, and to divide the cohort into two groups. This threshold was considered evaluating the definition of normal testosterone serum levels in most of guidelines published on the topic [34, 41–43]. Difference between the two sub-groups was evaluated using Mann–Whitney *U*-test.

Bivariate correlation analyses were performed by Spearman’s Rho, considering hormones and semen analysis parameters. The Bonferroni post-hoc test was applied to adjust statistical significance. Since 14 variables were considered in the correlation analysis, *p* < 0.003 was considered as statistically significant.

Multivariate stepwise linear regression analysis was applied, using testosterone serum levels as continuous dependent variable and all hormones and semen analysis parameters as independent ones. These analyses were performed tow times. The second one was performed adjusting the statistical models for confounders, such cryptorchidism, varicocelectomy, number of comorbidities, use of drugs, smoke and alcohol habits.

Two logistic regression analyses were performed using the number of men with testosterone serum levels below 3.5 ng/mL as dependent variable. The first analysis was applied considering clinical characteristics as independent variables, the second hormones and semen analysis parameters.

The IBM ® SPSS ® Statistics software for Windows (version 28.0.1.1; IBM SPSS Inc, Chicago, IL) was used for statistical analyses. Statistical significance was considered for *P* < 0.05.

## Results

Two hundred and fourteen men with idiopathic infertility were included among cases (mean age 38.2 ± 6.2 years) (Table 1). Two hundred and twenty-four subjects with normozoospermia were included among controls (mean age 33.7 ± 7.5 years).

**Table 1:** Clinical characteristics of men enrolled in the study. Data are expressed as median and interquartile range (IQR)

Variables	Cases (*n* = 214)
Age (years)	38.4 (6.9)
Body mass index (kg/m^2^)	27.9 (5.7)
Infertility duration (years)	2.6 ± 3.1 (1.0, 25.0)
Primary infertility *n* (%)	188 (72.6)
Anamnestic cryptorchidism *n* (%)	14 (5.4)
Varicocelectomy *n* (%)	18 (6.9)
Actual smokers *n* (%)	97 (37.5)
Number of cigarettes/daily	17.7 ± 8.6 (10, 40)
Ex smokers *n* (%)	45 (17.4)
Alcohol intake *n* (%)	135 (52.1)
Comorbidities	
Hypertension *n* (%)	14 (5.4)
Diabetes mellitus *n* (%)	3 (1.2)
Dislipidemia *n* (%)	2 (0.8)
Major cardiovascular events *n* (%)	1 (0.4)
Chronic disease score (CDS)	0.3 ± 1.1 (0, 8)
Current drugs assumption *n* (%)	46 (17.8)
Number of drugs	0.1 ± 0.5 (0, 6)
Anti-hypertensive *n* (%)	13 (5.0)
Lipid lowering *n* (%)	4 (1.5)
Anti-diabetic *n* (%)	2 (0.8)
Psyco-active *n* (%)	3 (1.2)

### Case-control comparison

According to the inclusion criteria, all patients enrolled among cases showed idiopathic infertility, thus with semen analysis parameters, below at least one of the WHO decisional limits (Table 2). In particular, 190 men (88.8%) showed a variable degree of semen parameters alterations, while 24 men (11.2%) were azoospermic. As expected in case of idiopathic infertility, gonadotropins serum levels resulted within reference ranges (Table 2). On the contrary, all subjects included in controls showed all semen analysis parameters above decisional limits (Table 2). Accordingly, both mean gonadotropins and testosterone serum levels were within the reference ranges (Table 2).

**Table 2:** Hormones and semen analysis results obtained during the diagnostic work-up for male infertility. Data are expressed as mean ± standard deviation (minimum, maximum)

Variables	Cases (*n* = 214)	Controls (*n* = 224)	*p*-value
Right testicular volume at orchidometer (mL)	14.6 ± 4.5 (5.0, 25.0)	—	—
Left testicular volume at orchidometer (mL)	14.7 ± 4.6 (5.0, 25.0)	—	—
Right testicular volume at ultrasound (mL)	15.7 ± 7.0 (5.7, 44.0)	—	—
Left testicular volume at ultrasound (mL)	14.1 ± 6.2 (4.3, 42.3)	—	—
Testosterone (ng/mL)	5.2 ± 2.0 (2.1, 14.5)	5.7 ± 1.5 (2.9, 11.0)	**0.002**
LH (IU/L)	4.0 ± 1.6 (1.4, 9.0)	3.8 ± 2.5 (1.0, 11.0)	0.324
T/LH	1.5 ± 0.9	1.9 ± 1.0	**<0.001**
FSH (IU/L)	5.2 ± 2.4 (1.0, 12.0)	4.1 ± 3.6 (1.0, 12.1)	**<0.001**
Estradiol (pg/mL)	24.8 ± 9.8 (9.0, 90.0)	—	—
Prolactin (ng/mL)	11.8 ± 5.6 (0.6, 21.7)	12.5 ± 8.0 (1.6, 48.6)	0.357
Semen volume (mL)	2.9 ± 5.6 (0.2, 11.5)	3.2 ± 1.6 (1.4, 9.0)	0.124
pH	8.1 ± 0.4 (6.0, 9.5)	8.1 ± 0.3 (7.0, 9.0)	0.905
Sperm concentration (million/mL)	8.8 ± 11.7 (0.0, 85.0)	93.5 ± 74.3 (16.5, 600.0)	**<0.001**
Total sperm number (millions)	20.6 ± 35.9 (0.0, 330.0)	267.7 ± 207.7 (41.4, 1512.0)	**<0.001**
Progressive motility (%)	18.8 ± 18.4 (0.0, 82.0)	53.6 ± 14.4 (30, 87)	**<0.001**
Total motility (%)	26.6 ± 22.5 (0.0, 100.0)	63.7 ± 13.4 (42, 96)	**<0.001**
Normal morphology (%)	2.0 ± 2.8 (0.0, 18.0)	10.0 ± 14.3 (4, 99)	**<0.001**

*FSH* follicle stimulating hormone, *LH* luteinizing hormone, *T* testosterone

Bold values indicate statistical significance

Interestingly, among cases, twelve patients (5.6%) were already under treatment with various antioxidants compounds at first andrology visit. Of this cohort, 190 men (88.8%) obtained a hormonal therapy prescription after the diagnostic work-up.

Cases and controls clearly showed significant different semen analysis parameters, as expected by inclusion criteria reported above (Table 2). Among hormonal variables, testosterone serum levels were significantly lower in cases compared to controls (*p* = 0.002) (Table 2). Although LH did not differ between cases and controls (*p* = 0.324), T/LH was significantly higher in controls than cases (*p* < 0.001). Moreover, FSH was significantly higher in cases compared to controls (*p* < 0.001) (Table 2).

### Total testosterone serum levels distribution

**Fig. 1:**
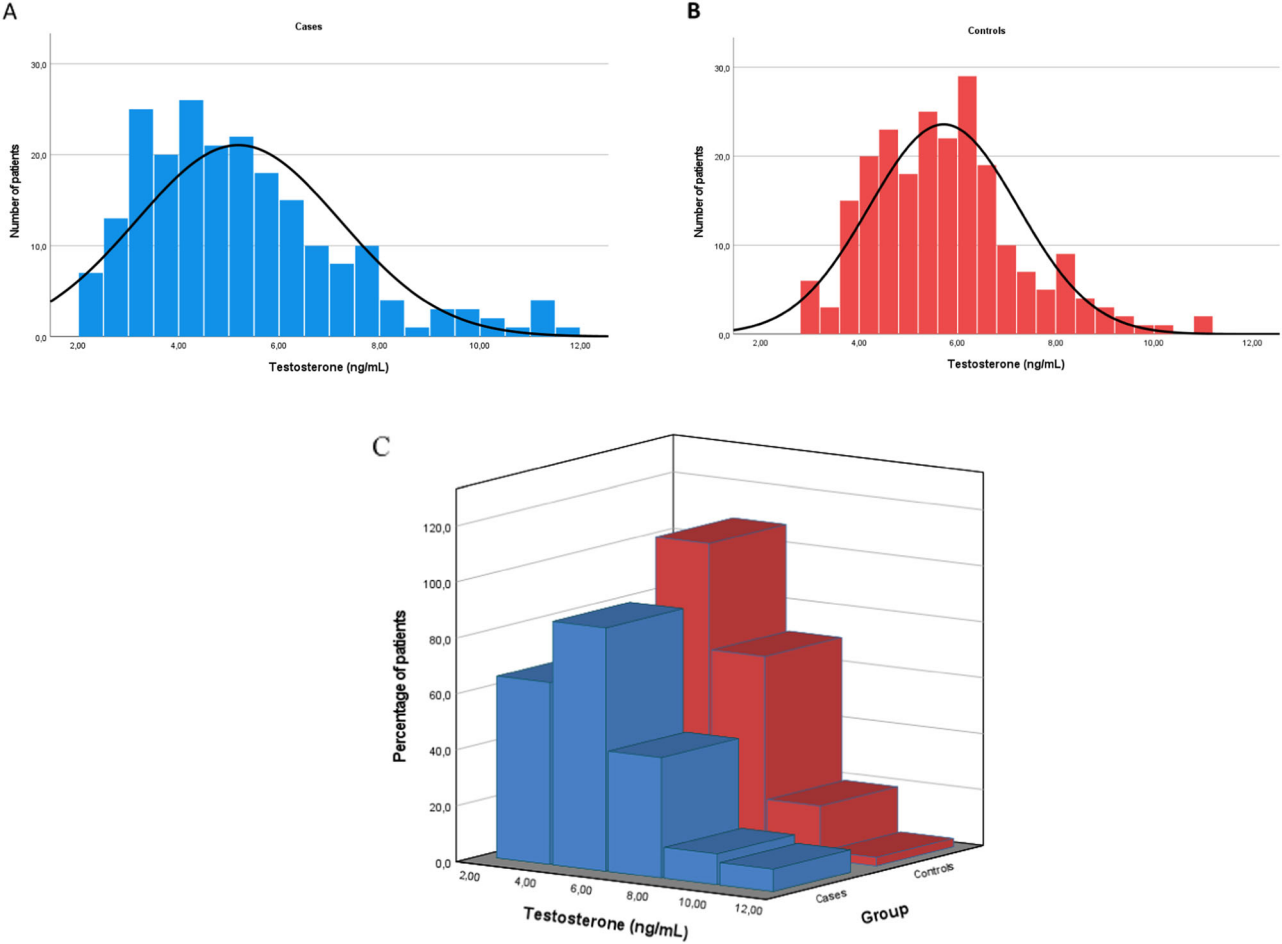
Total testosterone serum levels distribution in the cohort of idiopathic infertile men (**A**), and controls (**B**). **C** shows together cases and controls

In cases, total testosterone serum levels were not normally distributed (Shapiro–Wilk 0.933, *p* < 0.001), with a positive asymmetric distribution (Curtosi 0.7, standard error 0.3), with 95% confidence interval (CI) 4.9, 5.5 ng/mL (Fig. 1A). Similarly, testosterone serum levels were not normally distributed in controls (Shapiro–Wilk 0.967, p < 0.001), confirming the same positive asymmetric distribution detected in cases, (Curtosi 0.5, standard error 0.3), with 95%CI 5.5, 5.9 ng/mL (Fig. 1B). However, upon comparing the two curves, it was observed that the distribution of testosterone more accurately mirrored the Gaussian distribution in controls than in cases (Fig. 1C).

The first logistic regression analysis using anamnestic data as independent variables did not generate a significant model able to predict total testosterone serum levels distribution (Chi-squared 6.5, *p* = 0.588). Similar results were obtained by a second logistic regression analysis, using hormones and semen analysis parameters as dependent variables (Chi-squared 128.9, *p* = 0.063). Accordingly, bivariate correlation analyses did not highlight significant relationships between total testosterone serum levels and both hormones and semen analysis parameters (Table 3). The multivariate stepwise linear regression analysis generated two significant models. The first one related testosterone to estradiol serum levels (R = 0.205, standard error 1.9, *p* = 0.035), the second testosterone to LH serum levels (R = 0.302, standard error 1.9, *p* = 0.007). After confounders adjustment, the two models remained statistically significant (estradiol: R = 0.389, standard error 2.1, *p* = 0.009, LH: R = 0.290, standard error 1.9, *p* = 0.020).

**Table 3:** Hormones and semen analysis parameters differences between men with total testosterone serum levels below or above the 3.5 ng/mL threshold in cases. Data are expressed in mean ± standard deviation

Variables	Testosterone < 3.5 ng/mL	Testosterone ≥ 3.5 ng/mL	*p*-value
Right TV at orchidometer (mL)	14.5 ± 4.4	15.0 ± 4.9	0.529
Left TV at orchidometer (mL)	14.5 ± 4.4	15.5 ± 5.0	0.261
Right testicular volume at ultrasound (mL)	15.2 ± 6.6	16.7 ± 8.8	0.322
Left testicular volume at ultrasound (mL)	14.1 ± 5.9	14.4 ± 7.3	0.878
LH (IU/L)	4.0 ± 1.6	3.7 ± 1.5	0.242
FSH (IU/L)	5.1 ± 2.3	5.3 ± 2.7	0.689
T/LH	0.9 ± 0.4	1.7 ± 0.9	**<0.001**
Estradiol (pg/mL)	25.6 ± 10.2	22.4 ± 8.3	0.055
Prolactin (ng/mL)	11.7 ± 5.6	12.3 ± 5.5	0.556
Semen volume (mL)	2.9 ± 1.7	2.9 ± 1.7	0.912
pH	8.1 ± 0.3	8.0 ± 0.5	0.137
Sperm concentration (million/mL)	9.0 ± 12.3	8.1 ± 9.6	0.618
Total sperm number (millions)	21.2 ± 39.3	18.8 ± 22.7	0.683
Progressive motility (%)	19.5 ± 18.1	16.9 ± 19.4	0.392
Total motility (%)	28.7 ± 22.2	20.4 ± 22.3	0.066
Normal morphology (%)	2.3 ± 3.2	1.3 ± 1.7	0.064

*FSH* follicle stimulating hormone, *LH* luteinizing hormone, *T* testosterone, *TV* testicular volume

Bold values indicate statistical significance

### Subgrouping analyses

Twenty-eight% of cases (51 patients) showed total testosterone serum levels lower than 3.5 ng/mL. On the contrary, only the 4.5% of controls (10 subjects) showed testosterone serum levels lower than 3.5 ng/mL. The rate of reduced testosterone serum levels was higher in cases than controls (*p* < 0.001).

Dividing cases according to total testosterone serum levels threshold of 3.5 ng/mL, no significant differences were detected for hormones and semen parameters between the two groups (Table 4). Analysing separately men presenting testosterone higher than 3.5 ng/mL and men with testosterone below this threshold, interesting results were obtained. Indeed, no significant correlations remained for men with total testosterone serum levels higher than 3.5 ng/mL (Table 4). On the contrary, in the subgroup of patients with testosterone <3.5 ng/mL, total testosterone serum levels showed significant direct correlation with the percentage of sperm with normal morphology (Table 4). The same result was confirmed applying multivariate stepwise linear regression analysis, using total testosterone serum levels as dependent continuous variable. In men with total testosterone serum levels higher than 3.5 ng/mL, only the correlation with LH serum levels was highlighted both in unadjusted (R = 0.293, standard error 1.7, *p* = 0.010) and adjusted models (R = 0.401, standard error 1.9, *p* = 0.005) Table 5. On the contrary, in men with testosterone serum levels lower than 3.5 ng/mL, only normal sperm morphology significantly entered both the unadjusted (R = 0.430, standard error = 0.3, *p* = 0.020) and the adjusted models (R = 0.390, standard error = 0.5, *p* = 0.025).

**Table 4:** Bivariate correlation analysis between hormones and semen analysis parameters in cases

		BMI	Testosterone	LH	FSH	Estradiol	Prolactin	Semen volume	Semen pH	Sperm concentration	Total sperm number	Progressive motility	Total motility	Typical forms
Age	Rho	0.040	−0.070	−0.096	0.117	−0.078	−0.057	−0.238	0.016	0.085	−0.034	−0.050	−0.065	0.016
	*p*-value	0.681	0.308	0.160	0.087	0.294	0.437	**0.001**	0.831	0.222	0.627	0.495	0.400	0.843
BMI	Rho		−0.253	−0.256	−0.118	0.163	−0.147	−0.068	0.050	0.050	−0.002	0.069	0.083	−0.039
	*p*-value		0.008	0.007	0.220	0.119	0.147	0.486	0.631	0.609	0.981	0.492	0.422	0.709
Testosterone	Rho			0.097	−0.061	0.169	0.073	0.028	0.025	0.029	0.000	0.059	0.135	0.120
	*p*-value			0.157	0.376	0.021	0.323	0.689	0.742	0.676	0.998	0.422	0.080	0.131
LH	Rho				0.456	0.094	0.075	0.059	0.082	−0.139	−0.102	0.056	0.100	0.048
	*p*-value				**<0.001**	0.203	0.310	0.398	0.274	0.047	0.146	0.446	0.194	0.550
FSH	Rho					−0.063	−0.008	0.000	0.016	−0.117	−0.132	0.025	0.066	0.003
	*p*-value					0.396	0.918	0.997	0.830	0.095	0.058	0.734	0.392	0.969
Estradiol	Rho						−0.017	−0.072	−0.072	−0.062	−0.074	0.012	−0.009	−0.016
	*p*-value						0.828	0.335	0.371	0.410	0.323	0.880	0.915	0.851
Prolactin	Rho							−0.109	0.126	0.045	0.033	0.081	0.092	0.059
	*p*-value							0.147	0.115	0.556	0.663	0.306	0.267	0.490
Semen volume	Rho								−0.099	−0.248	0.135	−0.095	−0.067	−0.086
	*p*-value								0.191	**<0.001**	0.054	0.203	0.387	0.282
Semen pH	Rho									0.065	0.039	0.223	0.259	0.197
	*p*-value									0.389	0.607	0.005	**0.001**	0.021
Sperm concentration	Rho										0.884	0.37	0.356	0.384
	*p*-value										**<0.001**	**<0.001**	**<0.001**	**<0.001**
Total sperm number	Rho											0.379	0.365	0.369
	*p*-value											**<0.001**	**<0.001**	**<0.001**
Progressive motility	Rho												0.926	0.433
	*p*-value												**<0.001**	**<0.001**
Total motility	Rho													0.492
	*p*-value													**<0.001**

*BMI* body mass index, *FSH* follicle stimulating hormone, *LH* luteinizing hormone

Bold values indicate statistical significance

**Table 5:** Bivariate correlation analysis between total testosterone and both hormones and semen analysis parameters, dividing the cohort according to total testosterone serum levels

		Age	BMI	LH	FSH	Estradiol	Prolactin	Semen volume	Semen pH	Sperm concentratiion	Total sperm number	Progressive motility	Total motility	Typical forms
Testosterone serum levels ≥ 3.5 ng/mL														
Testosterone	Rho	−0.024	−0.137	0.064	−0.091	0.163	0.181	0.024	−0.128	−0.032	−0.088	−0.059	−0.017	−0.051
	*p*-value	0.762	0.232	0.419	0.249	0.054	0.031	0.765	0.139	0.691	0.272	0.492	0.854	0.595
Testosterone serum levels < 3.5 ng/mL														
Testosterone	Rho	0.068	−0.200	0.080	0.115	−0.356	0.137	0.140	0.280	0.162	0.235	0.271	0.180	0.338
	*p*-value	0.635	0.274	0.575	0.423	0.018	0.380	0.331	0.073	0.260	0.097	0.057	0.243	**0.002**

## Discussion

This real-world data analysis highlights the endocrine heterogeneity of men who are commonly classified within the broad diagnosis of ‘idiopathic infertility’. Indeed, whether these men uniformly show altered semen analysis and gonadotropin serum levels within reference ranges, testosterone serum levels are variable in this population. Twenty-four% of idiopathic infertile men show testosterone serum levels not frankly pathological, but in the ‘grey zone’ (i.e., between 2.1 and 3.5 ng/mL - 7.3 and 12.1 nmol/L), especially considering the age range of such patients. In other words, approximately a quarter of patients classified as idiopathic infertile present some sort of functional hypogonadism.

Our case-control study shows that testosterone serum levels are lower in subjects with altered spermatogenesis compared to those with normozoospermia. Our cases of idiopathic infertility show a significant reduction in all semen analysis parameters, together with higher FSH serum levels and reduced T/LH ratio compared to controls. The higher FSH serum levels detected in cases suggests an impairment of the spermatogenic compartment of the testis. The reduced T/LH ratio confirmed the reduced gonadotropin efficacy on testicular function, indirectly suggesting a potential spermatogenic impairment [40]. Thus, although our control group is not formed by men with proven fertility, it represents a valuable example of normal spermatogenesis. When testosterone serum levels distribution is evaluated in these groups, an asymmetric trend is described, showing a prolonged tail towards higher values. This distribution closely resembles that observed in normozoospermic patients; however, in the latter group, the distribution is closer to a Gaussian distribution. Moreover, the rate of subjects with reduced testosterone serum levels is higher in infertile men compared to normozoospermic subjects, confirming the relevance of evaluating hormonal profile in the diagnostic work-up of male infertility.

The International Committee for Monitoring Assisted Reproductive Technologies (ICMART) defined hypogonadotropic hypogonadism as the condition characterized by gonadal failure, i.e., impaired gametogenesis and gonadal steroid production, due to reduced gonadotropin production and/or action [44]. While reduced testosterone serum levels reflect reduced LH stimulation on Leydig cells, altered spermatogenesis is the result of the impairment of intra-testicular testosterone levels and/or FSH stimulation on Sertoli cells. Since this condition is caused by suboptimal testicular stimulation by gonadotropins, patients with hypogonadotropic hypogonadism could be effectively treated with exogenous gonadotropins or gonadotropin-releasing hormone (GnRH) with a significant testosterone raise and semen analysis improvement [45–47]. Alongside hypogonadotropic hypogonadism, testicular stimulation with gonadotropins could have other fields of application. For example, patients with not-obstructive azoospermia obtained a higher success rate of sperm retrieval by testicular sperm extraction (TESE) when they received hCG therapy (odds ratio [OR]: 1.295, 95%CI: 1.115–1.505; *p* < 0.001) [48]. Although definitive conclusions are not possible due to highly heterogeneous study designs, populations, sample sizes, gonadotropin therapy regimens, treatment duration and sperm retrieval methods, it is interesting to note that treatment with a hormone presenting steroidogenic function [49], i.e., hCG, is able to stimulate spermatogenesis. However, it is reasonable to think that a synergic action is exerted by both gonadotropins on testicular function, and thus, when testosterone serum levels are reduced, a replacement therapy with gonadotropins, e.g., hCG and FSH, could improve the chances of final success. However, the total testosterone serum levels threshold facilitating optimal spermatogenesis has yet to be established [50, 51]. With this in mind, our study suggests that 24% of men classified within the male idiopathic infertility category could benefit from gonadotropins’ stimulation. This is what currently occurs in clinical practice in Italy, where FSH administration is allowed. Here, FSH could be administered to men with idiopathic infertility presenting FSH serum levels below 8 IU/L [52]. This national rule allows the FSH prescription at the dosage of 150 IU three times weekly for four months, potentially renewable until 12 months of treatment. However, the expected FSH scheme is the same irrespective whether the patient shows hypogonadotropic hypogonadism or idiopathic infertility. Which should be the rationale suggesting that two different categories would benefit from the same therapeutic scheme? The male infertility treatment is still stuck in the 90 s, when fixed FSH dosages (i.e., 150 IU daily) were used even in ovarian stimulation, regardless of the characteristics of the woman. In this setting, now it is widely demonstrated that an over-stimulation is needed, and the personalization of the treatment is mandatory [53–55]. Thus, there is no rationale supporting the belief that all idiopathic men would respond to the same treatment, knowing that this category is highly heterogeneous. Here we suggest that, probably, the current treatment schedule could have a rationale only in 24% of men with idiopathic infertility.

On the other hand, our results suggest that the remnant 76% of men with idiopathic infertility showed normal testosterone serum levels, excluding a potential, functional hypogonadotropic hypogonadism form. Thus, these patients should not be treated with the same hormonal therapy scheme used to replace gonadotropins’ function, since the impaired spermatogenesis is probably caused by a defect within the spermatogenesis cascade. Obviously, further studies aimed to identify the aetiology behind the idiopathic forms of infertility are mandatory. However, considering the available knowledge, we can suggest that total testosterone serum levels could be used in idiopathic infertile men to decide whether the hormonal stimulation should be performed with a replacement or an over-stimulatory aim. Indeed, men with idiopathic infertility but normal pituitary gland stimulation on the testis probably will not respond to FSH administration at a replacement dosage. In the literature, 21 trials evaluated the efficacy of FSH administration in this setting, showing an overall pregnancy rate increase, although a high number-need-to-treat (NNT) was highlighted (from 10 to 18 men should be treated with FSH to obtain one pregnancy) [56]. This elevated NNT reflects the lack of rationale behind the current FSH scheme applied to idiopathic infertility. Indeed, whether 150 IU three times weekly should be sufficient in hypogonadotropic hypogonadism, it is probably underdosed in idiopathic infertility. Accordingly, a dose-dependent FSH-related sperm concentration increase is demonstrated by the comprehensive analysis of published data [7]. Moreover, several lines of research suggested that spermatogenesis does not run at its maximal level physiologically. Indeed, both animal and human models of hemicastration show a FSH and inhibin B increase with a consequent volume increase of the remaining testis, allowing a preserved (or just slightly reduced) spermatogenesis [57–61]. Thus, when the FSH serum levels increase, spermatogenesis could be boosted over its physiological level. This is further suggested by other human models, such as pituitary FSH-secreting adenomas [62], and activating *FSHR* mutations [63, 64]. There are no endocrinological reasons why the increase of a pituitary gland hormone should not be accompanied by the increased activity of the target gland.

Several limits need to be taken into consideration when evaluating our results. From one side, a real-world approach has some intrinsic limitations, since it works on retrospective, routinely collected data and there is not any a priori study design. Moreover, considering the study design, we included subjects in whom a definitive cause of infertility was ruled out. This implies that a subset of subjects, albeit limited, may exhibit conditions such as cryptorchidism, varicocele, and other factors potentially associated with infertility, even if not clinically demonstrated. In addition, testosterone serum levels measurements were performed using immunometric assays, that could be less accurate - especially for low values—if compared to the gold standard method, i.e., liquid chromatography-mass spectrometry [65]. Moreover, the testosterone serum level threshold selected to classify patients is not demonstrated to be the most accurate to discriminate eugonadal/hypogonadal patients, although supported by available literature. Finally, subjects enrolled among controls exhibited normal semen analysis parameters, yet their fertility status has not been conclusively established. It is crucial to note that when comparing infertile men to fertile subjects, only those individuals with documented fertility should be regarded as a suitable control group.

In conclusion, here we detect a subgroup of men with idiopathic infertility that, potentially, will better respond to an over-stimulatory FSH administration [66], accounting for the 76% of the entire group. Clearly these hypotheses require ad hoc-designed study to potentially optimize the use of FSH therapy and possibly increase its efficacy.
